# Continuous controllable balloon dilation: a novel approach for cervix dilation

**DOI:** 10.1186/1745-6215-13-196

**Published:** 2012-10-22

**Authors:** Slobodan Arsenijevic, Gordana Vukcevic-Globarevic, Vladislav Volarevic, Ivan Macuzic, Petar Todorovic, Irena Tanaskovic, Milan Mijailovic, Sasa Raicevic, Branislav Jeremic

**Affiliations:** 1 Clinic of Gynecology and Obstetrics, Clinical Center Kragujevac, Faculty of Medicine, University of Kragujevac, 69 Svetozara Markovica Street, Kragujevac, 34 000, Serbia; 2 Clinic of Gynecology and Obstetrics, Clinical Centre of Montenegro, bb Ljubljanska Street, Podgorica, 81 000, Montenegro; 3 Center for Molecular Medicine & Stem Cell Research Faculty of Medicine, University of Kragujevac, 69 Svetozara Markovica Street, Kragujevac, 34 000, Serbia; 4 Center for Terotechnology, Faculty of Mechanical Engineering, University of Kragujevac, 6 Sestre Janjic Street, Kragujevac, 34 000, Serbia; 5 Department of Histology, Faculty of Medicine, University of Kragujevac, 69 Svetozara Markovica Street, Kragujevac, 34 000, Serbia; 6 Clinic of Radiology, Clinical Center Kragujevac, Faculty of Medicine, University of Kragujevac, 69 Svetozara Markovica Street, Kragujevac, 34 000, Serbia

**Keywords:** Medical device, Balloon dilation, Cervix

## Abstract

**Background:**

Cervical dilation using mechanical dilators is associated with various complications, such as uterine perforation, cervical laceration, infections and intraperitoneal hemorrhage. To achieve safe and painless cervical dilation, we constructed a new medical device to achieve confident mechanical cervical dilation: a continuous controllable balloon dilator (CCBD).

**Methods:**

Controlled pumping of incompressible fluid into the CCBD increases the pressure and outer diameter of the CCBD, continuously dilating the cervical canal. The reliability of the CCBD was confirmed *in vitro* (testing for consistency and endurance, with no detected risk for breakage) and *in vivo.* A multi-center clinical study was conducted,with 120 pregnant women randomly assigned to one of three groups: Group I,control group, no dilation;Group II,mechanical dilation, Hegar dilator (HeD); and Group III,CCBD. The tissue material for histological evaluation was obtained from the endocervical mucosa before and after dilation using the HeD or CCBD.

**Results:**

The CCBD dilations were successful and had no complications in all 40 patients of Group III. The cervical tissue was markedly less damaged after CCBD dilation compared with HeD dilation (epithelium damage: 95% (HeD) vs. 45% (CCBD), *P* <0.001; basal membrane damage: 82.5% (HeD) vs. 27.5% (CCBD), *P* <0.001; stromal damage: 62.5% (HeD) vs. 37.5% (CCBD), *P* <0.01). Cervical hemorrhagia was observed in 90% of the patients after HeD dilation versus in 32.5% of the patients after CCBD dilation.

**Conclusions:**

The CCBD should be used as a replacement for mechanical dilators to prevent uterine and cervical injury during cervical dilation.

**Trial registration:**

ISRCTN54007498

## Background

Although cervical dilation is most commonly reserved for childbirth, its use has expanded to a large number of diagnostic procedures (dilation and curettage for diagnosing endometrial cancer, endometrial biopsies, evaluating the causes of infertility and hysteroscopy) and therapeutic procedures (cervical stenosis, dysfunctional uterine bleeding and dysmenorrhea, inserting intrauterine devices and draining the uterine cavity) [1-3]. Mechanical instruments, such as the Hern, Hegar, Pratt, Hanks and Denniston dilators, are used to sequentially dilate the cervix [4,5] by incrementally increasing the diameter of the inserted dilator until the dilation procedure is complete. However, these mechanical dilators require the use of appropriate force, which could permanently damage the cervical tissue and have adverse long-term effects on fertility [6-9]. Their use is associated with various potential complications, such as uterine perforation, cervical laceration, infections and intraperitoneal hemorrhages [4,5,10].

Several lines of evidence have suggested that cervical priming, accomplished by pharmacological agents (prostaglandin analogs), can prevent cervical laceration by reducing the force required for cervical dilation [3,10]. The main disadvantage of pharmacological agents is that the woman might experience bleeding and cramping prior to the surgical procedure [11-13].

To achieve safe and painless cervical dilation, we constructed a medical device for reliable mechanical cervical dilation: a continuous controllable balloon dilator (CCBD). The CCBD is based on a patented solution [14] that combines all of the advantages of traditional mechanical devices with several new features that give the provider full process control in all of the phases of cervical dilation. We conducted a pilot study that showed a significant reduction in all of the side effects related to dilator use for the CCBD compared with metal mechanical devices.

## Methods

### Study organization

The study (ISRCTN54007498) was conducted at the Gynecology & Obstetrics Clinics at Kragujevac Clinical Center, Serbia, and Podgorica Clinical Center, Montenegro. The data were collected by the study coordinators at the participating centers. The authors vouch for the completeness and accuracy of the data and analyses. An independent data and safety monitoring board monitored the study and reviewed the protocol compliance and outcome data. The protocol was approved by each participating center's institutional review board.

### Study patients

The patients were enrolled in the study using the following criteria: age between 19 and 40; pregnancy verified by an ultrasound; singleton pregnancy; gestational age ≤10; uterus and cervix with normal findings; and absence of uterine contractions or bleeding.

Patients were excluded from the study if any of the following criteria were met: any signs of spontaneous abortion; any previous attempt at an abortion or use of substances for cervical maturation; multiple pregnancy; the presence or, at minimum, the suspicion of a septic abortion, followed by an elevated body temperature of 38°C or higher, uterine pain and odorous vaginal secretions; the presence of any previous intervention performed on the uterine cervix; uterine or cervical anomalies; an intrauterine device *in situ*; or hemorrhagic and/or chronic diseases.

### Study design

The study included 120 pregnant women randomly assigned to one of the following three groups: Group I (40 pregnant women), control group with no dilations; Group II (40 pregnant women), dilations performed using Hegar dilators (HeDs); and Group III (40 pregnant women), dilations performed using the CCBD.

The experiments were undertaken with the understanding and appropriate informed consent of each patient.

### Continuous controllable balloon dilator: main characteristics

**Figure 1:**
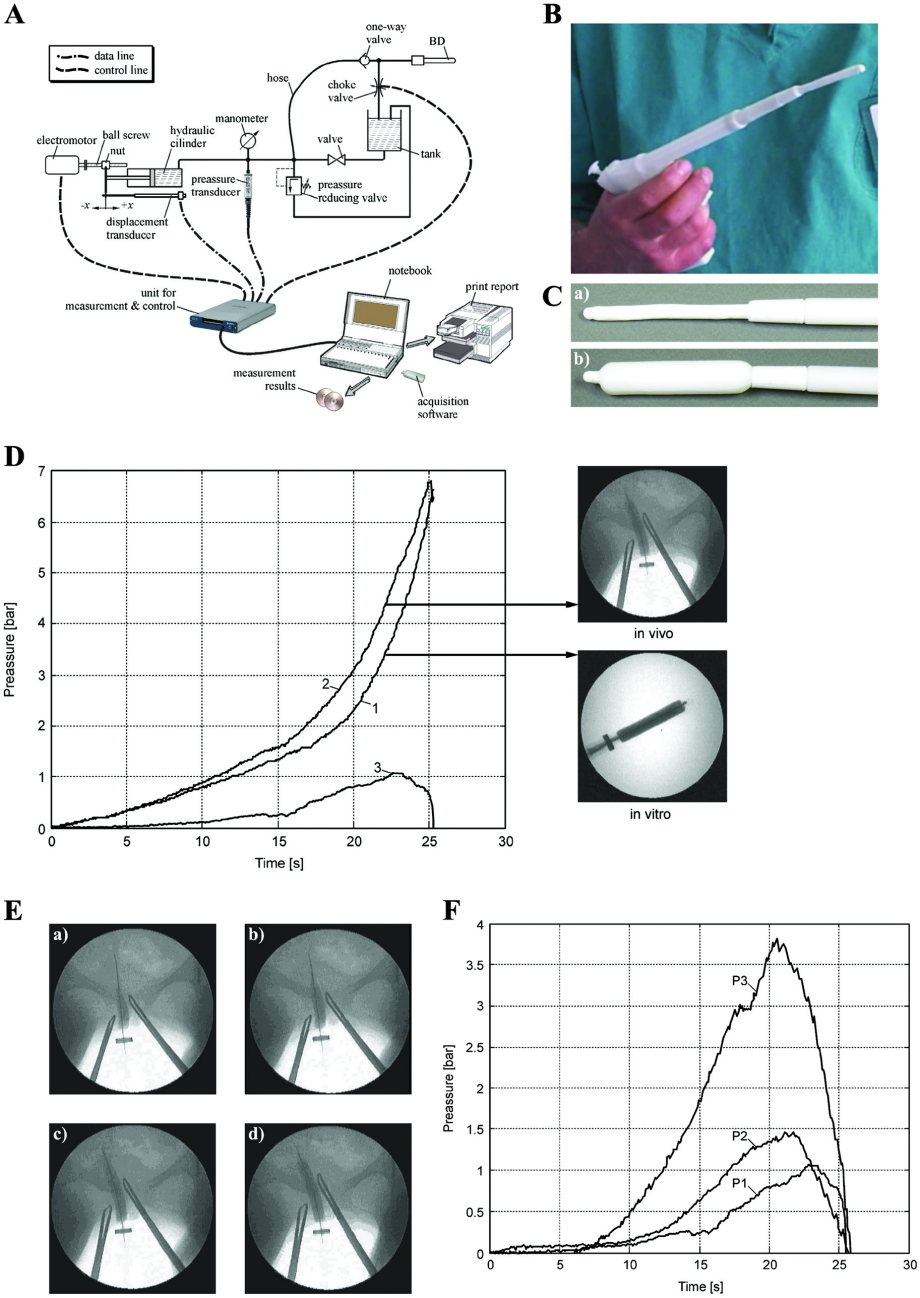
**System for continuous controllable balloon dilation.** (**A**)The continuous controllable balloon dilator (CCBD) system for cervical dilation. (**B**) Image of the CCBD. (**C**) CCBD with an uninflated BD (a) and an inflated BD (b). (**D**) The calculation of cervical resistance during cervical dilation using the CCBD: line 1, change in pressure during *in vitro* balloon dilation; line 2, change in pressure during *in vivo* cervical dilation using the CCBD; line 3,difference in the change in pressure between the *in vivo* and *in vitro* experiments, which represents the resistance of the cervical tissue to CCBD dilation. (**E**) Pictures of the phases of *in vivo* cervical dilation using the CCBD (after 10, 15, 20 and 25 seconds). (**F**) The comparative results of CCBD cervical dilations for three representative patients: P1, the cervical resistance of Patient 1 was depressed after 23 seconds with a pressure of 3.8 bars; P2, the cervical resistance of Patient 2 was depressed after 22 seconds with a pressure of 1.4 bars; P3, the cervical resistance of Patient 3 was depressed after 21 seconds with a pressure of 1.1 bars.

The CCBD is a fully controllable device for cervical dilation (Figure 1A) based on the use of a specially constructed balloon dilator (BD) that consists of three layers: an inner silicone layer, a central layer made from high-strength fabric, and an outer silicone layer (Figure 1B,C). The maximum BD expansion diameter is limited by the central layer. The outer silicone layer contacts the cervical tissues during dilation. The consistency and endurance of this BD were tested at a pressure of 25 bars, with no detected risk for breakage. The reliability of the CCBD was confirmed *in vitro* and *in vivo* (Figure 1D, 1E).

Dilation using the CCBD is performed continuously, with only one dilator placement. In this study, the CCBD was integrated into a system that enables real-time data acquisition and the monitoring of the parameters relevant to the biophysics of dilation (Figure 1A). Dilation dynamics directly depend on the flow of an incompressible fluid into the BD, which is an easily controllable parameter. Because it is an incompressible working fluid, distilled water was used with the addition of nonionic contrast medium (Ultravist-300; Schering AG, Berlin, Germany), which enabled the visual monitoring of dilation using a digital subtraction apparatus for angiography (Figure 1A).

Incompressible fluid from the hydraulic cylinder is pumped to the BD via a flexible hose (hose) and one-way valve (Figure 1A). Fluid flow is controlled by an electric motor that, via a spiral spindles/nut system, provides constant speed of the hydraulic cylinder piston and consequently constant fluid flow. The actual position of the hydraulic cylinder piston and the fluid pressure are monitored by displacement and pressure transducers. The unit for measurement and control collects signals from those two transducers. The pressure gauge is used for visual control of the current fluid pressure, while the pressure reduction valve represents a safety element. After finalization of the dilatation procedure, the choke valve is opened by remote command, which results in draining of fluid from the BD. Then the BD could be easily extracted from the cervical canal. Dilatation duration and maximum pressure in the BD represents parameters assigned by a PC-controlled unit for measurement and control. Those parameters could be monitored in real-time parameters by PC and appropriate acquisition software, which enable full controllability of the cervical canal dilatation process (Figure 1A).

### Histological evaluation

Tissue material for the histological evaluation of cervical damage was obtained from the endocervical mucosa by single curettage (Novac Curette, CooperSurgical, Trumbull, Connecticut, USA) before and after dilation using the HeD or CCBD. The samples were stained with H&E and blinded analysis was performed using a light microscope (Olympus BX 51; Olympus, Hamburg, Germany). The surface areas of the hemorrhagic regions were measured according to a previously published protocol [15].

### Statistical analysis

All of the statistical analyses were conducted using SPSS 13.0 for Windows (IBM, New York, New York, USA). The results were analyzed using Student’s *t* test. All of the data in this study are expressed as the mean ± standard error. *P* < 0.05 was considered significant.

## Results

### Continuous controllable balloon dilation results

The CCBD dilations were successfully performed on all patients. The providers and patients reported no complications or problems with the CCBD, a new dilation technique. The average duration of CCBD dilation was approximately 25.5 seconds (Figure 1F) and was independent of the individual patient's cervical resistance (Figure 1F). The maximal recorded pressure in the BD during dilation was significantly lower than the test pressure for BD consistency and endurance.

### Comparative analysis of biophysical phenomena during cervical dilation by HeD and CCBD

**Figure 2:**
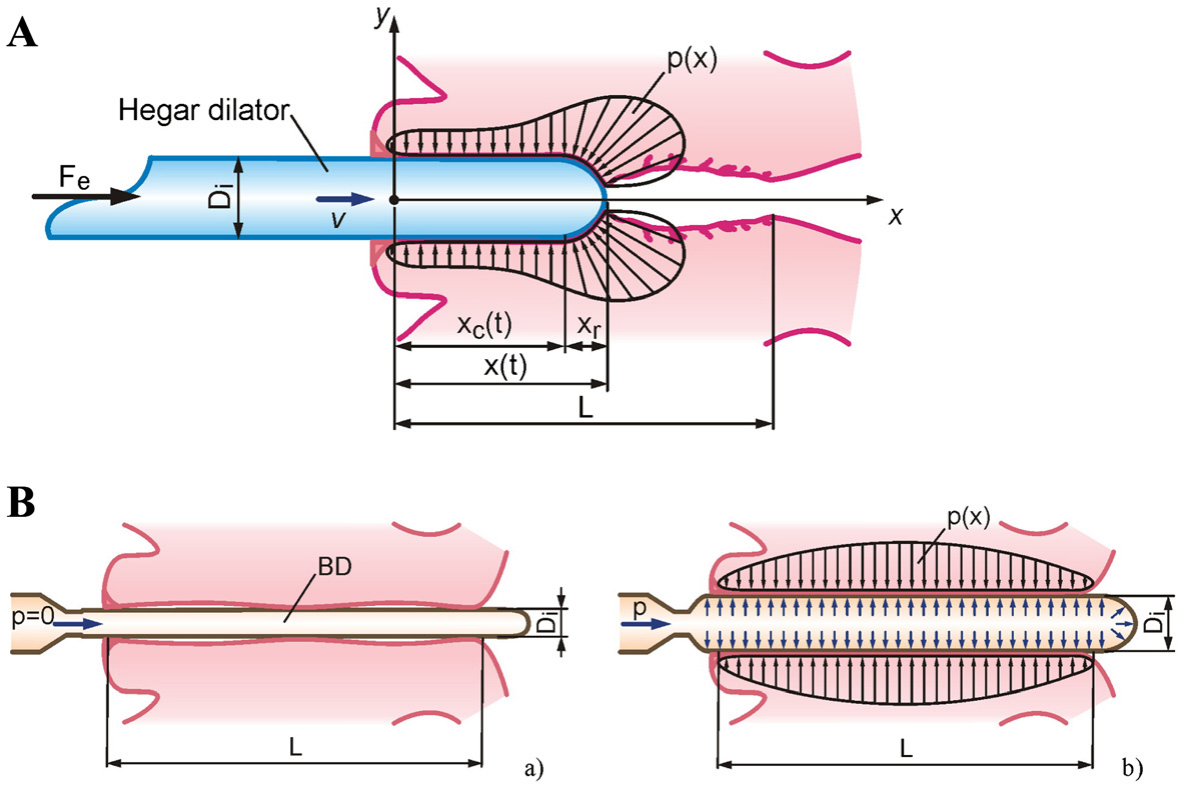
**Biophysical phenomena during cervical dilations.** (**A**) Biophysical model of Hegar dilator (HeD) dilatation. Under external force *F*_*e*_ (required to dilate the cervix at each stage of dilation), the dilator of diameter *D*_*i*_ moves at speed *v* through the cervical canal having length *L*. *F*_*e*_ must overcome the resultant sum of forces that appeared in the direction of HeD movement. During dilation with the N^0^9 HeD (diameter *D*_*i*_ = 9 mm) the mean recorded value of external force ${\overset{―}{F}}_{e}$was 11 N (with vaginal sodium nitroprusside (SNP) gel) and 17 N (with vaginal misoprostol) [A]. The mean recorded ${\overset{―}{F}}_{e}$ during dilation with the N^0^10 HeD (diameter *D*_*i*_ = 10 mm)was 13 N [B]. The mean ${\overset{―}{F}}_{e}$ required to complete cervical canal dilation points to the complex biophysics of processes in the contact zone of tissue and the HeD. HeD motion along the cervical canal (length *L*) in the direction of the internal uterine os leads to changes in geometry of the cervical canal. Tissue, in different ways, opposes this change of geometry in contact with the HeD, characterized by the pressure distribution *p*(*x*). (**B**) Biophysical model of balloon dilatation (BD) by continuous controllable balloon dilator (CCBD). (a) Initial BD form diameter is approximately*D*_*i*_ ≈ 4.5 mm,enabling insertion into the cervical canal with very low resistance to penetration. (b) Pumping of incompressible fluid in the BD leads to increased pressure and outer diameter of the BD, causing dilatation of the cervical canal. The dilatation process is performed simultaneously on the entire length (*L*) of the cervical canal, where relative movement (sliding) between the tissue/BD contact pair is almost reduced to zero. In this case the cervix tissue opposes less the change in geometry characterized by *p*(*x*).

The basic biophysical models of cervical canal dilation using the HeD and CCBD are shown in Figure 2. Under the action of an external force *F*_*e*_ (the force required to dilate the cervix at each stage of HeD cervical dilation), the HeD (with diameter *D*_*i*_) moves at speed *v* through the cervical canal (with length *L*). The external force *F*_*e*_ must overcome the resulting sum of the forces that appear in the direction of the moving HeD. The HeD motion leads to changes in the geometry of the cervical canal. In various ways, the tissue opposes these geometrical changes upon contact with the HeD, which is characterized by the pressure distribution *p*(*x*). Because a relatively small zone of the cervical canal is affected by dilation at this time, high contact loads are produced in the tissue (Figure 2A). A detailed overview of the basic forces involved in the contact zones between the HeD and cervical canal tissue during dilation indicates that only one component of the applied external force is responsible for dilation. None of the other forces directly participate in dilation and may lead to a large number of side effects.

In contrast, the CCBD dilation procedure involves inserting the BD in its initial form (diameter *D*_*i*_ ≈ 4.5 mm) into the cervical canal, which results in a very low resistance to penetration (Figure 2B). Pumping incompressible fluid into the BD increases the outer diameter and pressure of the BD, which dilates the cervical canal. Dilation is performed synchronously along the entire length *L* of the cervical canal, where the relative movement between the tissue/BD contact pair is reduced to almost zero. In this case, the cervical tissue also opposes changes in its geometry, which is characterized by a much more uniform pressure distribution *p*(*x*) (Figure 2B). All of the components of the involved forces participate in dilation, which significantly reduces any side effects.

### Cervical tissue is markedly less damaged and the extent of cervical hemorrhagia is significantly lower after CCBD compared with HeD dilation

**Figure 3:**
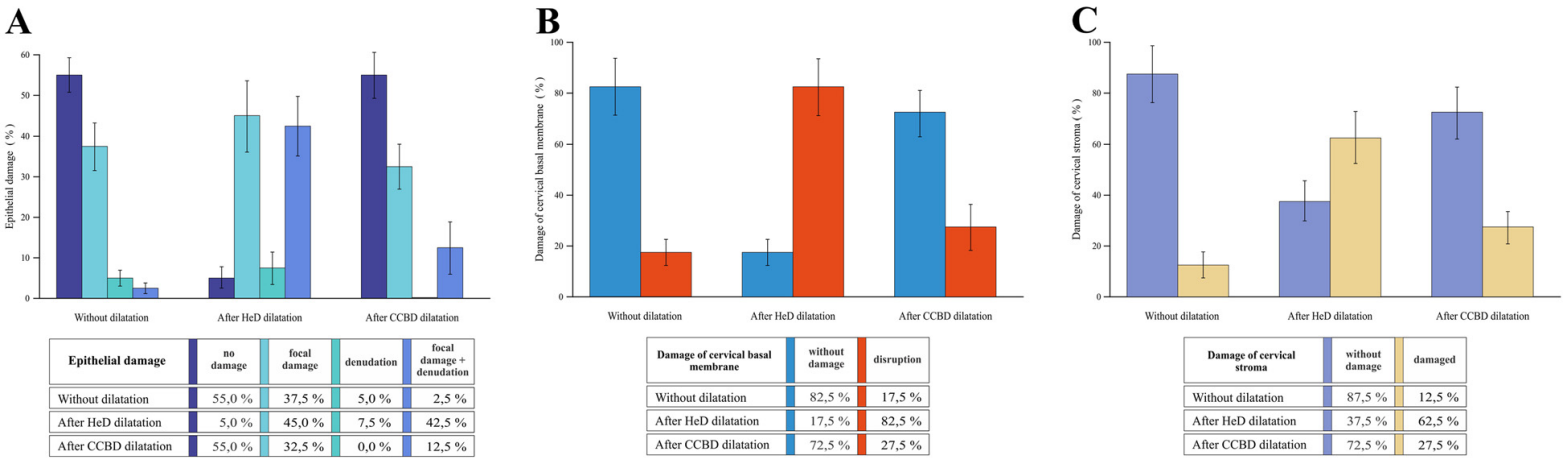
**Cervical tissue damage after Hegar dilator and continuous controllable balloon dilator dilation.** (**A**) Percentage of patients with cervical epithelial damage. There was a statistically significant difference in the percentage of epithelial damage between the cervices dilated using the Hegar dilator (HeD) and the cervices dilated using the continuous controllable balloon dilator (CCBD). Mean ± standard error, *n* = 40 per group, *P* <0.001. (**B**) Percentage of patients with cervical basal membrane damage. There was a statistically significant difference in the percentage of basal membrane damage between the cervices dilated using the HeD and the cervices dilated using the CCBD. Mean ± standard error, *n* = 40 per group, *P* <0.001). (**C**) Percentage of patients with cervical stromal damage. There was a statistically significant difference in the percentage of stromal damage between the cervices dilated using the HeD and the cervices dilated using the CCBD. Mean ± standard error, *n* = 40 per group, *P* < 0.001).

There was a statistically significant difference in the percentage of epithelial damage (*P* <0.001), basal membrane damage (*P* <0,001) and stromal damage (*P* <0.01) when comparing cervices dilated by the HeD with cervices dilated by the CCBD (Figure 3).

**Figure 4:**
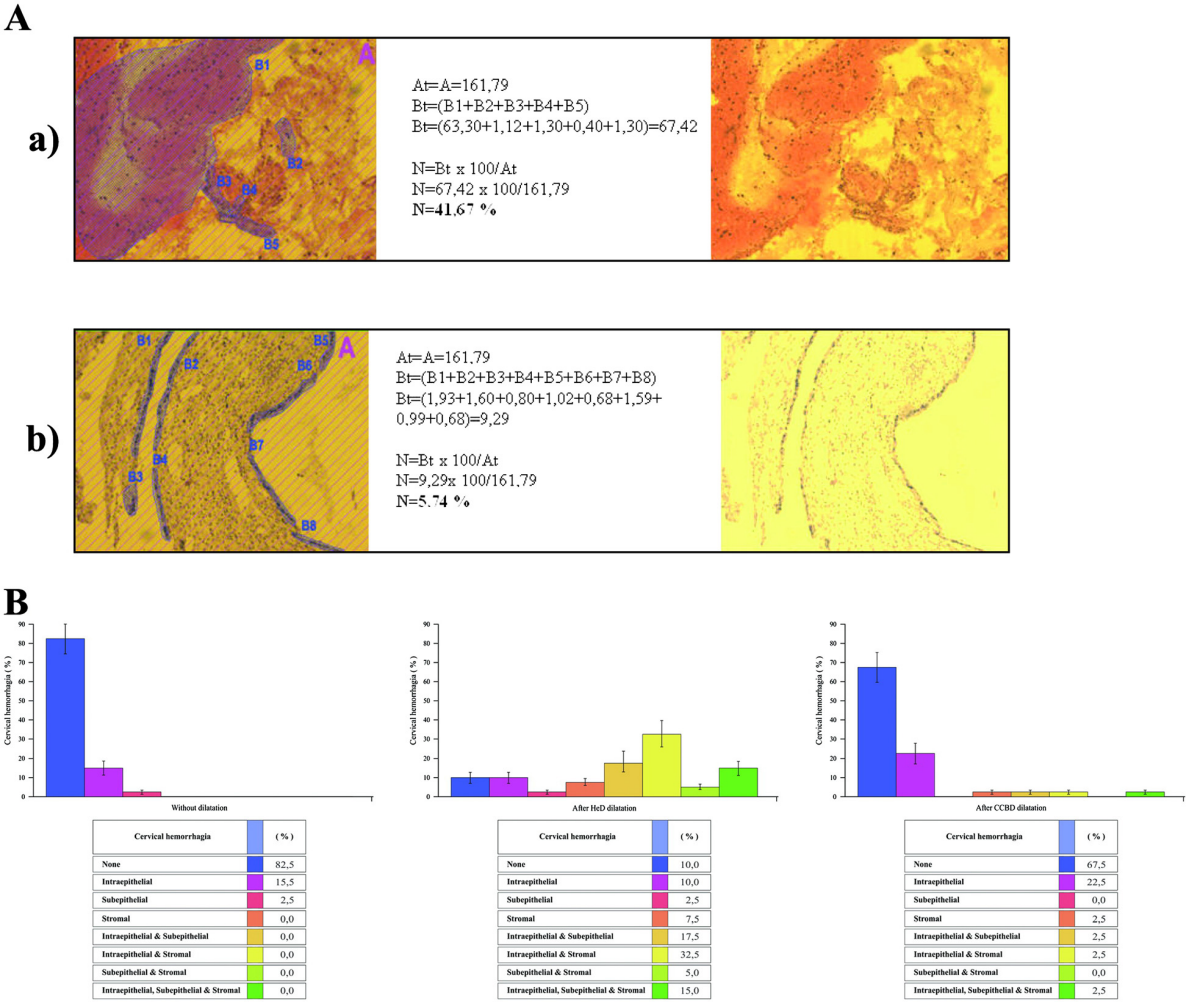
**Extent of cervical hemorrhagia after Hegar dilatorand continuous controllable balloon dilator dilation.** (**A**) A semiquantitative determination of representative samples. Intraepithelial, subepithelial and stromal bleeding was observed after Hegar dilator (HeD) dilation (a) compared with intraepithelial hemorrhagia observed after continuous controllable balloon dilator (CCBD) dilation (b). (**B**) Percentage of patients with cervical hemorrhagia after HeD and CCBD dilation. A significant difference in the extent of cervical hemorrhagia was observed between the nondilated and HeD-dilated patients. Mean ± standard error, *n* = 40 per group, *P* <0.01. There was no significant difference in the extent of cervical bleeding observed between the nondilated and CCBD-dilated patients. The extent of cervical hemorrhagia was significantly lower after CCBD dilation compared with HeD dilation. Mean ± standard error, *n* = 40 per group, *P* <0.01.

In addition, the extent of cervical hemorrhagia was significantly lower (*P* <0.01) after CCBD dilation compared with HeD dilation (90% after HeD dilation vs. 32.5% after CCBD dilation; Figure 4).

## Discussion

Based on our results, CCBD dilation is a novel, non-invasive, fully controllable and safe clinical procedure; and compared with the predominant mechanical method for cervical dilation (HeD) [4], CCBD is a less invasive and more reliable system for cervical dilation.

The main factors leading to uterine and cervical injury during dilation procedures that use metal mechanical dilators are the provider's inexperience, an abnormal uterine cavity and/or an anteflexed or retroflexed uterus [13,16-19]. In addition, adolescents have a higher risk for cervical laceration because they usually have small, physiologically immature cervices that are difficult to dilate [20-22]. Another significant problem with current cervical dilators is a lack of control over the rate of dilation – extremely rapid cervical dilation often leads to cervical laceration [23,24].

All of the aforementioned risk factors (the provider's inexperience, the lack of control over the rate of dilation, differences in uterine position and cervical immaturity) could be overcome using the CCBD. Dilation with the CCBD is fully controllable, safe and independent of the provider's experience. In the case of incompressible fluid BD breakage (which did not happen during our study), the leakage of only a few drops of fluid results in a sudden pressure drop in the BD; therefore, the risk for patient injury in this case is almost non-existent. Both the trainees and the experienced providers could use the CCBD without the risk for cervical laceration or uterine perforation. It is noteworthy that CCBD cervical dilation was successfully performed on all of the patients, regardless of the patient's age, cervical stenosis, uterine position and previous obstetric history. In addition, no side effects were observed during the procedure or after successful cervical dilation. However, it is important to note that due to limited number of patients enrolled in this study, a large prospective study should be conducted in order to confirm the effectiveness of CCBD dilation.

## Conclusions

We suggest the CCBD as a replacement for mechanical dilators, with the main purpose of preventing uterine and cervical injury during cervical dilation. Depending on the opinion of the physician, the CCBD could be used alone or in combination with cervical priming agents.

In the end, it should be emphasized that CCBD dilation is a new, original, non-invasive, fully controllable and safe technique for cervical dilation. However, because of the limited number of patients enrolled in this study, a large prospective study should be conducted in the future to further confirm the effectiveness of CCBD dilation.

## Abbreviations

BD: Balloon dilator; CCBD: Continuous controllable balloon dilator; H&E: Hematoxylin and eosin; HeD: Hegar dilator.

## Competing interests

The authors state that they have no competing interests.

## Authors’ contributions

SA was aninventor of the CCBD, designer of the study and principal investigator, and was responsible for data collection and interpretation, manuscript editing and final approval of the manuscript. GV-G was responsible for research, data collection and interpretation. VV was responsible for conception and design of the manuscript, manuscript writing and data interpretation. IM was responsible for research, manuscript writing and editing. PT was responsible for research, manuscript writing and editing. IT was responsible for data collection and interpretation. MM was responsible for research, data collection and interpretation. SR was responsible for research, data collection and interpretation, and manuscript editing. BJ was an inventor of the CCBD and designer of the study, and was responsible for manuscript editing. All authors read and approved the final manuscript.

## Ethics approval

Institutional approval for the study was granted by the Ethics Committee of the Kragujevac Clinical Center and the Ethics Committee of the Podgorica Clinical Center in accordance with internationally accepted ethical standards (the Helsinki Declaration of 1964, last revised in 2005). The experiments were undertaken with the understanding and appropriate informed consent of each patient.
